# Influence of inflammation on the expression of microRNA-140 in extracellular vesicles from 2D and 3D culture models of synovial-membrane-derived stem cells

**DOI:** 10.3389/fbioe.2024.1416694

**Published:** 2024-08-07

**Authors:** João Pedro Hübbe Pfeifer, Fernanda de Castro Stievani, Célio J. da Costa Fernandes, Gustavo dos Santos Rosa, Emanuel Vitor Pereira Apolonio, Mariana Correa Rossi, Willian Fernando Zambuzzi, Ana Liz Garcia Alves

**Affiliations:** ^1^ Regenerative Medicine Lab, Veterinary Surgery and Animal Reproduction Department, School of Veterinary Medicine and Animal Science, São Paulo State University - UNESP, Botucatu, Brazil; ^2^ Biophysics and Pharmacology Department, Institute of Biosciences, São Paulo State University - UNESP, Botucatu, Brazil; ^3^ Laboratory of Bioassays and Cellular Dynamics, Department of Chemical and Biological Sciences, Institute of Biosciences, São Paulo State University - UNESP, Botucatu, Brazil

**Keywords:** articular homeostasis, equine, exosomes, microRNA, osteoarthritis, nanotechnology, translational medicine

## Abstract

**Background:**

In osteoarthritis (OA), articular homeostasis is regulated by microRNA-140 that inhibits ADAMTS-5, an enzyme that cleaves aggrecan and stimulates the synthesis of other inflammatory mediators. This study aims to evaluate the expression of microRNA-140 in extracellular vesicles (EVs) derived from equine synovial-membrane-derived mesenchymal stem cells (eqSMMSCs) cultured in monolayer (2D) and three-dimensional (3D) culture models under an *in vitro* inflammatory environment.

**Methods:**

Four experimental groups of eqSMMSC cultures were defined for isolation of the EVs. The 2D and 3D control groups were cultured in a conventional cell culture medium, while the 2D-OA and 3D-OA treatment groups were exposed to an OA-like medium containing IL-1β and TNFα. The culture media samples were collected at 24 h, 72 h, and 120 h time points for EV isolation and characterization using nanoparticle tracking analysis (NTA) and transmission electron microscopy (TEM). Reverse transcription quantitative polymerase chain reaction was employed to assess the expressions of microRNA-140 in both the cells and EVs. All statistical analyses were conducted at the 5% significance level.

**Results:**

Encapsulation of the eqSMMSCs protected the cells from the inflammatory media compared to the monolayer cultures. EVs were found in higher concentrations in the 3D-OA cultures. Additionally, higher expressions of microRNA-140 were observed in the cells of the 3D-OA group at 24 and 72 h, whereas microRNA-140 expressions in the EVs were higher in the 3D group at 72 h and in the 2D-OA group at 120 h (*p* < 0.001). However, the 3D-OA culture showed higher expression of the mRNA *Adamts5* in the EVs at 120 h.

**Conclusion:**

The responses of the eqSMMSCs to inflammatory stimuli involve intracellular expression of microRNA-140 and its subsequent transportation via the EVs, with quicker responses observed in the 3D than 2D cultures. This study sheds light on the behaviors of stem cells in restoring homeostasis in osteoarthritic joints.

## Introduction

Several studies have described the action mechanisms and functions of mesenchymal stem cells (MSCs), including their paracrine actions and capability for differentiation (Carvalho et al., 2014; Caplan, 2017; Li et al., 2017), thereby establishing their potential as a source of bioactive factors for tissue repair (Cassano et al., 2018a; Lange-Consiglio et al., 2018a; Park et al., 2019; Ragni et al., 2020). The paracrine actions of MSCs involve mechanisms mediated by extracellular vesicles (EVs) that are released into the extracellular space and subsequently internalized by the target cells. These EVs act as carriers of functional molecules that exert paracrine effects (Ratajczak et al., 2006; Camussi et al., 2010; Lange-Consiglio et al., 2018a). This mechanism provides avenues for drug-delivery research by leveraging the natural capabilities of EVs to encapsulate molecules and their tropism to reach the affected sites (Crivelli et al., 2017).

MicroRNAs are small fragments of endogenous RNAs transported in the EVs. Despite being non-coding components, microRNAs play crucial roles in regulating the expressions of various genes involved in cell regulation and homeostasis (Miyaki and Asahara, 2012). Cell communications are often mediated through the delivery of microRNAs encapsulated in EVs to influence mRNA expressions in the target cells (Kosaka et al., 2010; Pegtel et al., 2010). This mechanism is observed across multiple diseases in various species, including humans and horses (Lange-Consiglio et al., 2018a; Qiu et al., 2018; Ragni et al., 2020). Scientific findings related to musculoskeletal disorders in experimental animal models, such as the equine (McIlwraith et al., 2012) and ovine species, can be transversely applied to human health. These animal models are particularly relevant for studying human orthopedic conditions, including osteoarthritis (OA), given that OA occurs naturally in these species (Kuyinu et al., 2016; Ribitsch et al., 2020).

OA is a multifactorial joint disease with a high incidence in both humans and horses (Frisbie et al., 2002). At the molecular level, osteoarthritic joints exhibit downregulation of certain microRNAs, which contribute to the progression and perpetuation of this disease (Iliopoulos et al., 2008; Miyaki and Asahara, 2012; Mobasheri et al., 2017; Budd et al., 2018). MicroRNA-140 exerts direct effects on articular homeostasis, demonstrating tissue-specific expressions and functions across various species like humans, horses, and mice (Iliopoulos et al., 2008; Miyaki et al., 2009; Nakamura et al., 2011; Buechli et al., 2013; van der Kolk et al., 2015; Yin et al., 2017). In the osteoarthritic microenvironment, there is a downregulation of microRNA-140 expression compared to healthy cartilage (Miyaki et al., 2009; Tardif et al., 2009; Zhang et al., 2021); this downregulation is attributed to an increase in the expression of the target mRNA *Adamts5* (a disintegrin and metalloprotease with thrombospondin type 5) (Miyaki et al., 2010). A*damts5* is a precursor of aggrecanase-2 or ADAMTS5, a metalloproteinase that plays a significant role in promoting signaling to inflammatory enzymes and proteins like IL-1β, thereby contributing to cartilage degradation (Kobayashi et al., 2013; Sandy et al., 2015). ADAMTS5 directly cleaves aggrecan, which is the molecule responsible for maintaining binding within the collagen network (Glasson et al., 2005).

According to Miyaki et al. (2009), stimulation with IL-1β leads to negative regulation of microRNA-140 and positive regulation of ADAMTS5. Conversely, transfection of microRNA-140 in chondrocytes not only enhances aggrecan synthesis but also suppresses ADAMTS5 expression induced by IL-1β (Miyaki et al., 2009). This evidence strongly supports that ADAMTS5 is a direct target of microRNA-140, indicating its pivotal chondroprotective role in cartilage metabolism (Miyaki et al., 2010; Karlsen et al., 2016; Tao et al., 2017; Yin et al., 2017; Duan et al., 2020; He et al., 2020). Cell-based therapies and regenerative medicine have been employed extensively to restore articular homeostasis. Bioengineering techniques play crucial roles in creating optimal conditions for the cells to perform their biological functions effectively. For instance, MSCs intended for use in chondral repair demonstrate improved proliferation and chondrogenic differentiation when cultured in three-dimensional (3D) sodium alginate scaffolds, which not only mimic the *in vivo* environment more closely but also allow enhanced molecular communication facilitated by the porosity of the alginate capsules (Santos et al., 2018; Santos et al., 2019).

MSCs sourced from various tissues offer strategies for treating different diseases in tissue engineering and regenerative medicine. Among these, adipose-tissue-derived MSCs (ADMSCs) and bone-marrow-derived MSCs (BMMSCs) are widely used owing to their easy availability and isolation. However, MSCs exhibit epigenetic memory, making it crucial to consider using cells from tissues closely related to the specific target of treatment or study, as this can significantly influence cellular signaling and functional properties (Walewska et al., 2023). Synovial-membrane-derived mesenchymal stem cells (SMMSCs) constitute another source that can be easily obtained through arthroscopy (Baboolal et al., 2018) and are promising for treating joint diseases. Studies have explored the use of these cells in OA models, including in rodents and equines for orthopedic research (Estakhri et al., 2020; Santos et al., 2023). *In vitro* studies have demonstrated that SMMSCs exhibit superior potential for chondrogenic differentiation compared to other cell sources (Yoshimura et al., 2007). Another critical aspect of SMMSCs is that when they are cultured in a 3D environment such as an alginate hydrogel, their potential for chondrogenic differentiation is enhanced, as shown in several studies (Sun et al., 2011; Lee and Mooney, 2012; Lee et al., 2013; Santos et al., 2018).

Therefore, studies investigating the responses of cells under different culture methods in an inflammatory environment provide crucial data and significantly contribute to the field of regenerative medicine. Understanding how such cells behave under these conditions is essential for developing effective therapeutic strategies aimed at treating inflammatory and degenerative conditions like OA. Such research can inform the optimization of culture techniques and development of targeted therapies that harness the regenerative potential of MSCs.

## Methods

The aim of this study was to characterize the EVs secreted by equine SMMSCs (eqSMMSCs) under two different culture methods and to evaluate the expressions of microRNA-140 within these vesicles for assessing the impacts of an inflammatory medium on these parameters. The present study was conducted in accordance with the principles of ethics and animal welfare in experimentation and has received approval from the Ethics Committee on Animal Use (CEUA) of the School of Veterinary Medicine and Animal Science of UNESP, Brazil (protocol number 187/2019). Additionally, the recommendations of the International Society for Extracellular Vesicles (Welsh et al., 2024) were adhered to throughout this study.

### Experimental design

The SMMSCs used in this study were obtained from our laboratory’s cell storage. These cells were derived from a 1.5-year-old quarter horse healthy donor without any clinical or infectious conditions relevant to the research objectives. Prior to this study, the SMMSCs were characterized for immunophenotype and trilineage differentiation capabilities, as described in a previously published work from our laboratory (Rosa et al., 2020).

For this study, two control and two treatment groups were defined. The control groups consisted of eqSMMSCs cultured in a monolayer (2D) and eqSMMSCs encapsulated in sodium alginate beads (3D), both of which were cultured in a conventional medium containing DMEM-F12/glutamax +10% fetal bovine serum (FBS) +1% antibiotic–antimycotic (penicillin + streptomycin + amphotericin B) (#10565018, #12657029, and #15240062, respectively, Gibco, Grand Island, NY, United States). The other two groups were subjected to an inflammatory medium termed “OA-like”; these groups included eqSMMSCs cultured in a monolayer (2D-OA) and eqSMMSCs encapsulated in sodium alginate beads (3D-OA), both of which were exposed to the OA-like medium containing IL-1β and TNFα.

All eqSMMSCs were initially cultured in a monolayer using culture flasks until reaching 80% confluency. The cells intended for the 3D and 3D-OA groups were subsequently encapsulated and incubated for 12 h to allow adaptation to the new 3D organization before introduction of the corresponding experimental medium. Each experimental group was cultured in triplicate at each time point to ensure robustness and reliability of the results. For the 24-h and 72-h time points in each group, 1.5 mL of the medium was used per well. At the 120-h time point, 1 mL of the medium was added after 72 h of cell culturing to ensure adequate nutrition without changing the medium.

**FIGURE 1:**
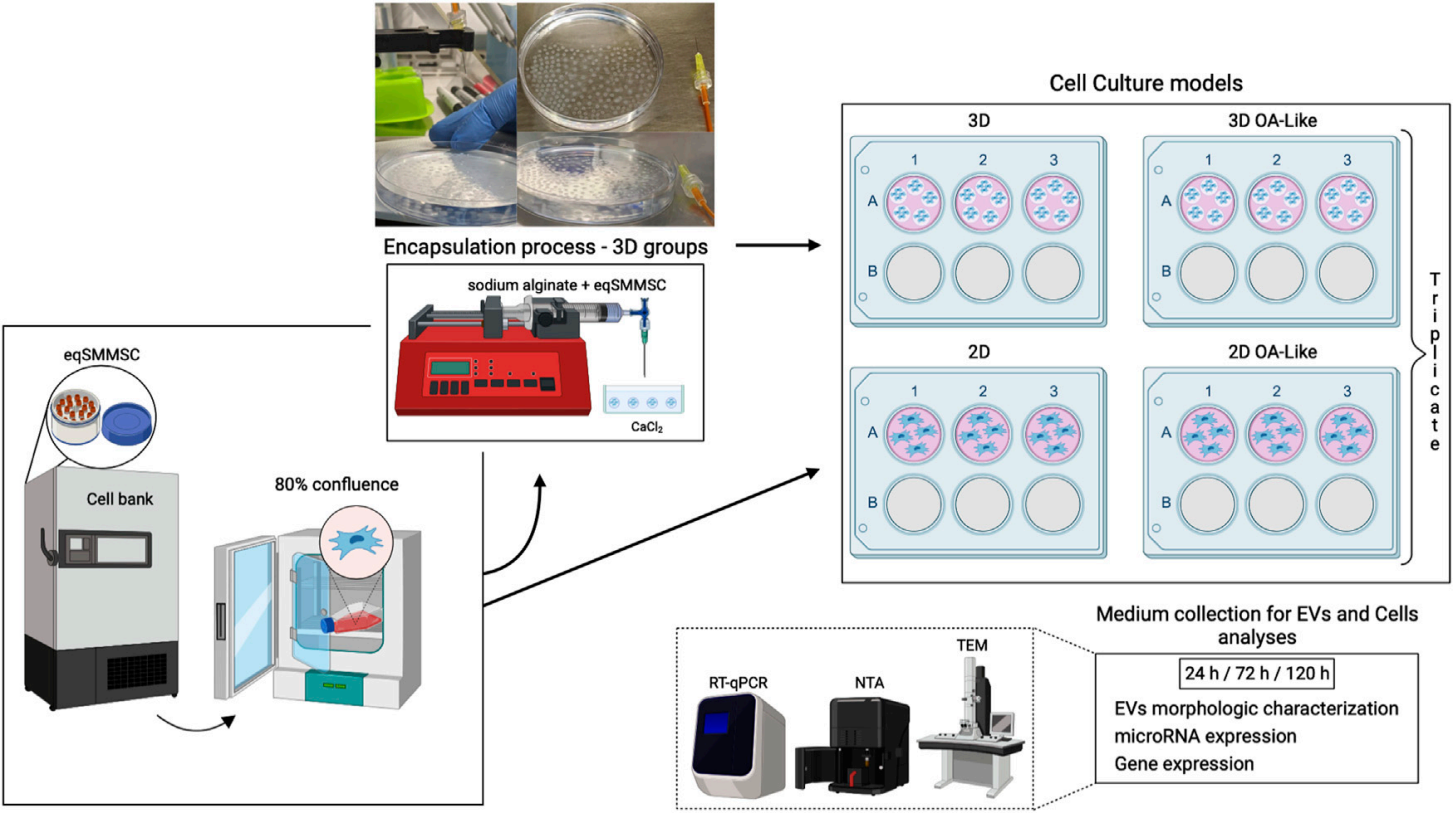
Study design. First, equine synovial-membrane-derived mesenchymal stem cells were cultured in a monolayer until reaching 80% confluency, after which they were counted for encapsulation (upper image of the encapsulation diagram) for the 3D groups; the remaining cells were counted for the 2D groups. Initially, all groups received a total of 2.5 × 10^5^ cells/well. A conventional cell culture medium was used in groups 2D and 3D; for groups 2D-OA and 3D-OA, we used an inflammatory cell culture medium (10 ng/mL of IL-1β and 0.25 ng/mL of TNF-α). For extracellular vesicle (EV) isolation, were define 24, 72, and 120 h as the experimental time points. The EVs were characterized for their morphologies, sizes, concentrations, and tetraspanin expressions. We also performed analyses of the microRNA-140 expressions on eqSMMSCs and EVs as well as expression of *Adamts5* on the EVs.

Samples of the culture medium were collected from all experimental groups at 24 h, 72 h, and 120 h after initial exposure, and the EVs were isolated from these media. The remaining cells at each time point were lysed directly on the culture plate for the 2D or 3D groups sodium alginate capsules were first dissolved by incubating in 4% sodium citrate for 20 min at 37°C; after dissolution, the cells were washed with phosphate buffer solution (PBS), centrifuged to collect the cell pellets, and then lysed using QIAzol lysis reagent (#217004, Qiagen, Germantown, MD, United States) following the manufacturer protocols. Following lysis, the samples from both the 2D and 3D groups were stored for subsequent analyses using reverse transcription quantitative polymerase chain reaction (RT-qPCR), as depicted in Figure 1.

### Depletion of EVs in FBS

FBS (#12657029, Gibco, Grand Island, NY, United States) and buffer solution were filtered with a 0.22-μm mesh and ultracentrifuged to remove the eventual xenogenic or contaminant EVs. Here, an ultracentrifuge was used (Sorvall Ultra Pro 80, DuPont, Wilmington, DE, United States) with a rotor for 30-mL tubes (T865 Sorvall, DuPont, Wilmington, DE, United States). The centrifugation was performed overnight at 120,000×*g* and 4°C to produce EV-free FBS (FBS*evfree*) that was stored at −20°C. The transmission electron microscopy (TEM) images of FBS*evfree* are shown in Supplementary Data S1.

### Encapsulation and experimental culture of eqSMMSCs

The cells were trypsinized at 80% confluence (Trypsin-EDTA 0.25%, #25200072, Gibco, Grand Island, NY, United States); after counting, 5 × 10^6^ cells were resuspended in 0.84 mL of 1.5% sodium alginate (#W201502. Sigma-Aldrich, Saint Louis, MO, United States) and placed in a 10 mL syringe attached to an infusion pump. The solution was dripped into 1.2 M CaCl_2_ and maintained for 20 min to allow alginate crosslinking. The newly formed capsules were placed in a 6-well plate. Each well received five capsules containing approximately 5 × 10^4^ cells each (2.5 × 10^5^ cells/well), which was the same number of starting cells/well as the 2D groups. The cell number and viability were not measured at any time point for the 3D and 2D cultures.

Each group was plated in triplicate, and all cultures were washed with PBS to remove any remaining media or CaCl_2_, followed by an adaptation time of 12 h in a culture medium containing DMEM-F12/glutamax +10% FBS*evfree* + 1% antibiotic–antimycotic (penicillin + streptomycin + amphotericin B), which had the same constitution as the medium used in the 2D and 3D groups subsequently. The OA-like medium was composed of DMEM-F12/glutamax +10% FBS*evfree* + 1% antibiotic–antimycotic +10 ng/mL IL-1β (Sylvester et al., 2012) (#PHC0814. Gibco, Grand Island, NY, United States) and 0.25 ng/mL TNFα (Westacott et al., 2000) (#PHC3015L. Gibco, Grand Island, NY, United States).

### Isolation of the EVs

Isolation protocols were performed using the *Total Exosome Isolation* kit (from cell culture media) (#44783559, Invitrogen, Carlsbad, CA, United States) according to the manufacturer’s instructions. Briefly, the sample media were centrifuged at 2,000×*g* for 30 min at 4°C, and the supernatants were placed in RNase-free tubes before mixing with the isolation solution in the ratio of 0.5:1 mL. The samples were homogenized and incubated overnight at 4°C in a stirrer plate. After centrifuging at 10,000×*g* for 1 h at 4°C, the supernatants were removed and the pellets containing the EVs were recovered with 200 μL of PBS*.* The samples were then stored at −80°C until analysis.

### Morphological characterizations of the EVs

The samples were fixed in nickel grids (Lässer et al., 2012), and the EVs were characterized according to their morphologies and sizes/concentrations by TEM (Philips CM200, 160 kV, filament LaB6). The mean size and concentration were determined by nanoparticle tracking analysis (NTA) using the NanoSight (NS300, Malvern Panalytical, ENG) equipment.

### Expression of microRNA

Quantitative analyses of microRNA-140 in the EVs and eqSMMSCs were performed by RT-qPCR, and all analyses were carried out following manufacturer recommendations. Total DNA extraction was performed with the *miRNeasy Mini* kit (#217004, Qiagen, Germantown, MD, United States). The cDNA reverse transcription of each sample was performed using the *TaqMan MicroRNA Assay Reverse Transcription* kit (#4366596, Applied Biosystems, Foster City, CA, United States), and microRNA-140 was detected using the *TaqMan Fast Advanced Master Mix* reaction kit (#4444556, Applied Biosystems, Foster City, CA, United States) and a *TaqMan microRNA Assay* probe (*mmu-mir-140-5p,* a commercial sequence homologous to the equine microRNA-140 *eca-mir-140-5p* (Buechli et al., 2013), confirmed by mirbase.org) (#assayID001187, Applied Biosystems, Foster City, CA, United States). The gene U6 snRNA *TaqMan microRNA Assay* (#assayID001973, Applied Biosystems, Foster City, CA, United States) was used as the internal control for normalization of the differences between the samples. The obtained data were analyzed using the 2^−ΔΔCT^ method (Livak and Schmittgen, 2001), and all steps were executed using the QuantStudio 3 (Applied Biosystems, Foster City, CA, United States) equipment.

### Gene expression

Quantitative analyses of *CD9*, *CD63*, *CD81*, and *Adamts5* within the EVs were performed by RT-qPCR from a pool of triplicate results of each group at each time point. The primers were obtained from Exxtend Biotecnologia (Paulínia, SP, BRA) and are described in Supplementary Data S2. Extraction of the DNA from the EVs for RT-qPCR was performed using Qiazol with the *miRNeasy Mini* kit (#217004, Qiagen, Germantown, MD, United States). The cDNA reverse transcription was performed with the *high-capacity cDNA reverse transcription* kit (#4368813 Applied Biosystems, Foster City, CA, United States). The GAPDH gene was used as the internal control for normalizing the differences among the samples, and the data were analyzed using the 2^−ΔΔCT^ method (Livak and Schmittgen, 2001). All steps were executed using the QuantStudio 3 equipment.

### Statistical analysis

Data on the microRNA expressions, gene expressions, sizes, and concentrations of the EVs were subjected to normality testing using the Kolmogorov–Smirnov test. The parametric data were analyzed using one-way ANOVA, while the non-parametric data were analyzed using the Kruskal–Wallis test. Tukey’s multiple comparisons test was used to compare the groups showing statistical differences. Additionally, Friedman’s repeated measures test was used to compare different time points within each group. Correlation analysis between the microRNA values of cells and EVs was conducted using Pearson’s correlation test (P), followed by linear regression to obtain the adjusted R-squared value. All statistical analyses were performed using GraphPad Prism software at the 5% significance level.

## Results

### Cell encapsulation

**FIGURE 2:**
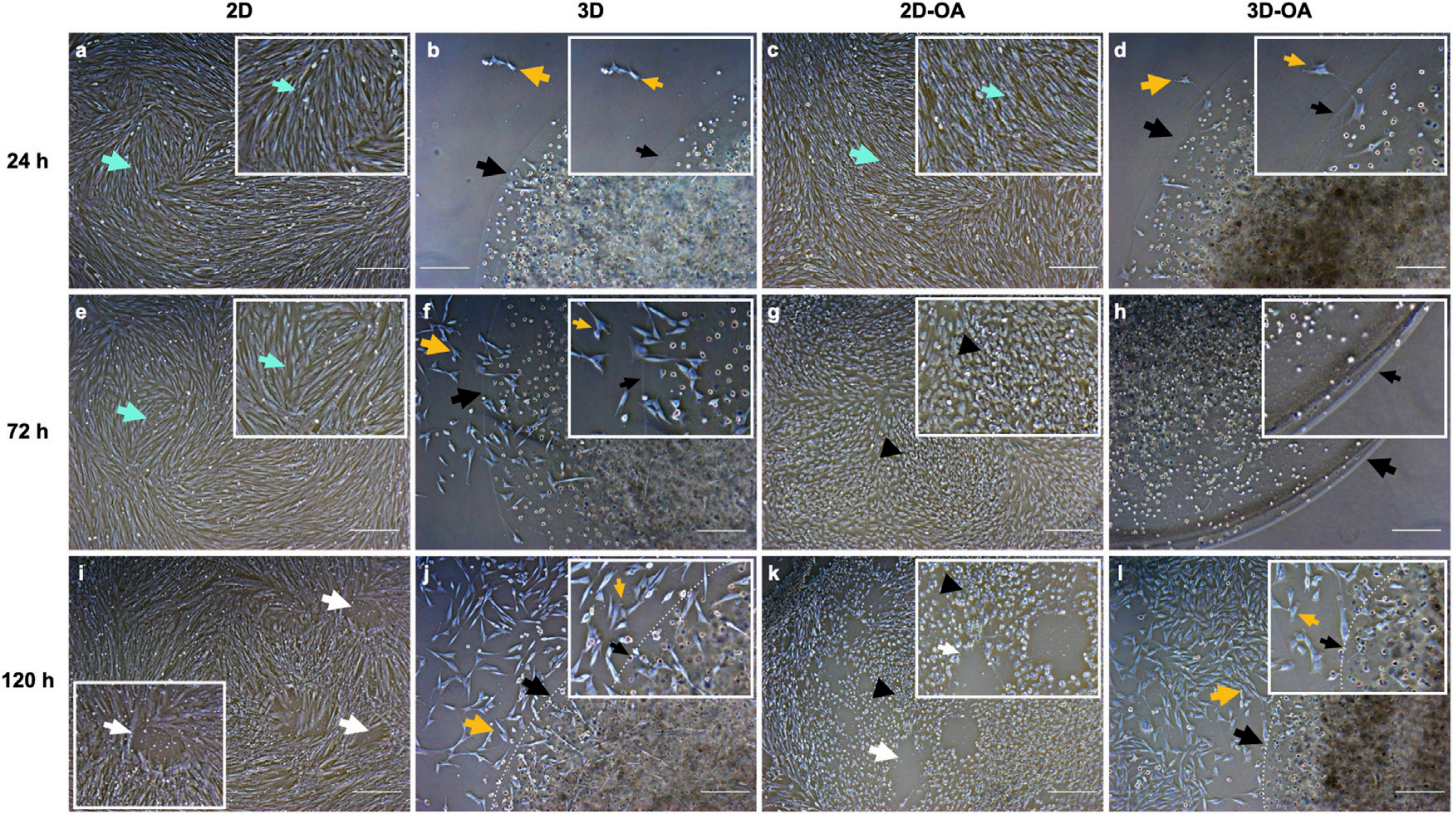
Comparisons between the cell cultures at the corresponding moments of conditioned medium collection. The boxed corners indicate the areas. **(A–D)** 24 h, **(E–H)** 72 h, and **(I–L)** 120 h. The green arrows indicate the fibroblastic morphologies of the eqSMMSCs **(A,C,E)**. The black arrows in **(B,D,F,G,H,J,L)** indicate the borders of the alginate hydrogel capsules, and the white dotted lines indicate the capsule borders in **(J,L)**. In **(B–D),** the yellow arrows indicate cell migration from the capsule to the plate and subsequent adherence to the plate, showing fibroblastic morphology; in **(F,G,H,J,L),** we observe adherence of the plate cells that migrated and/or proliferated as well as adherence of the eqSMMSCs under the capsule to the plate. The white arrows in **(I,K)** indicate areas of repelled eqSMMSCs, given the high confluence. Cells with loss of the fibroblastic morphology (black arrowheads) are indicated in **(G,K)**. Magnification ×5 and scale bar 200 µm.

Several differences were noted among the cell cultures (Figure 2). Cells from the 2D group maintained a fibroblastic morphology and high confluence, whereas cells from the 2D-OA group showed retraction of the filopodial bundles after 24 h, losing the fibroblastic morphology and adopting a cuboidal shape with low confluence at the subsequent time points, indicating cell death. The cells of the 3D group remained within the alginate capsules after 24 h, showing initially low rates of migration/proliferation that increased to a large value at 120 h, as demonstrated by the attachment of the cells to the well surface. Similarly, the 3D-OA group showed higher cell confluence and attachment at 120 h.

### Isolation of the EVs

**FIGURE 3:**
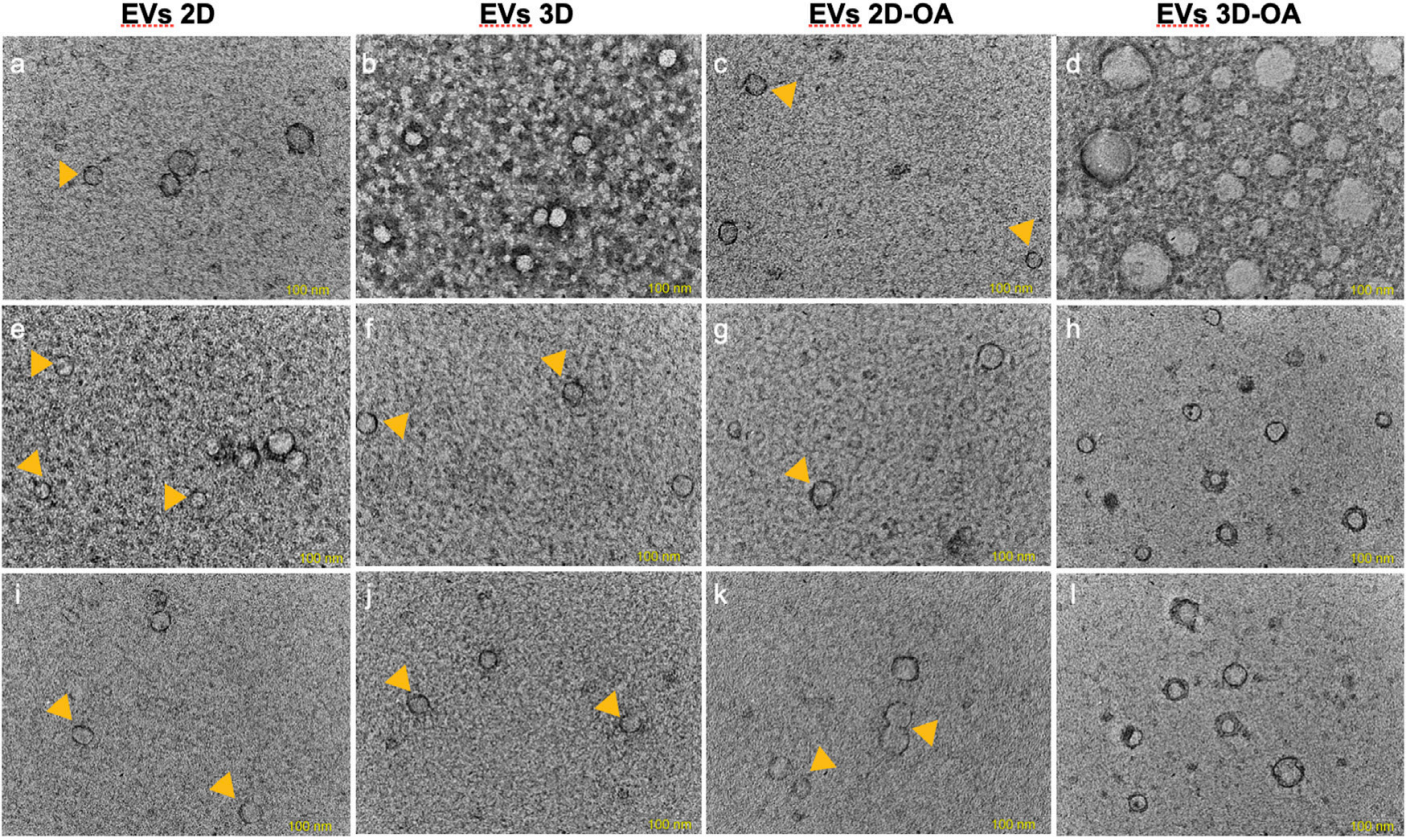
Transmission electron microscopy. The images correspond to **(A–D)** 24 h, **(E–H)** 72 h, and **(I–L)** 120 h. Small EVs are indicated by the yellow arrowheads, and the lipid bilayers of the EVs are observed in **(A,D,G,H,K,L)**. Scale bar 100 nm.

**FIGURE 4:**
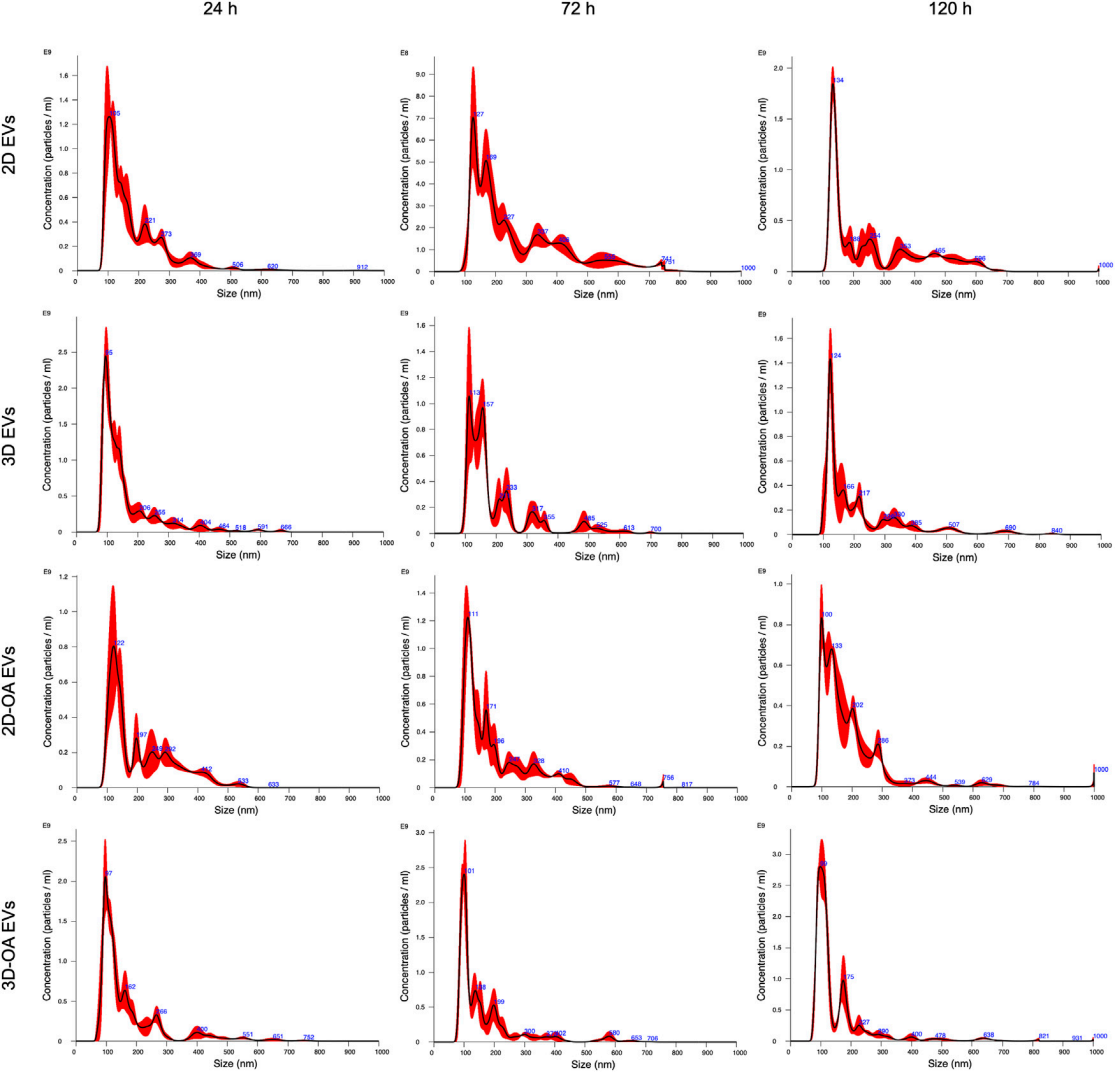
Nanoparticle tracking analysis showing the concentration and average size at each time point.

The method of isolation was successful in all groups and time points, as observed in the TEM (Figure 3) and NTA (Figure 4) results. Analyses of the concentration and size of the EVs demonstrated variations between groups and time points (Table 1, 2, respectively). The concentration of EVs in the 3D group was similar to that for the 3D-OA group at 24 h but higher than those of the 2D and 2D-OA groups. The 3D-OA group was statistically similar to the 2D group but had higher value than the 2D-OA group, which presented with the lowest concentration of EVs among all groups. Group 3D-OA presented with a statistically higher concentration of EVs compared to all groups at 72 h (*p* = 0.006) and 120 h (*p* < 0.001). Comparing the time points for the 3D-OA group, there were no differences at the 24 h and 72 h time points, but a significant increase in concentration was observed at 120 h (*p* = 0.037).

**TABLE 1:** Nanoparticle tracking analysis. EV concentration is in terms of particles/mL × 10^11^. The data are presented as mean ± SD.

Groups	Time points			*p* value
	24 h	72 h	120 h	
2D	1.24 ± 0.09 ^bA^	0.90 ± 0.17 ^bB^	1.27 ± 0.02 ^bA^	0.046
3D	1.64 ± 0.02 ^aA^	0.93 ± 0.06 ^bB^	0.82 ± 0.08 ^cB^	<0.001
2D-OA	0.81 ± 0.13 ^cA^	1.03 ± 0.16 ^bA^	0.86 ± 0.12 ^cA^	0.210
3D-OA	1.46 ± 0.22 ^abB^	1.36 ± 0.03 ^aB^	1.79 ± 0.07 ^aA^	0.037
*p* value	<0.001	0.006	<0.001	

^a^ ^,b,A,B,c^Mean values followed by the same lowercase letters along the columns and uppercase letters along the rows did not statistically differ based on Tukey’s test (*p* > 0.05).

**TABLE 2:** Nanoparticle tracking analysis of mean EV sizes (nm) for different groups and culture models. The data are presented as mean ± SD.

Groups	Time points			*p* value
	24 h	72 h	120 h	
2D	180.97 ± 17.37 ^abB^	272.53 ± 16.70 ^aA^	268.33 ± 16.06 ^aA^	0.006
3D	160.37 ± 1.14 ^bB^	198.43 ± 23.5 ^bA^	219.53 ± 15.10 ^bA^	0.010
2D-OA	214.17 ± 17.69 ^aA^	196.10 ± 20.05 ^bA^	188.13 ± 3.63 ^cA^	0.182
3D-OA	174.77 ± 7.65 ^bA^	167.27 ± 18.73 ^bA^	155.40 ± 1.76 ^dA^	0.316
*p* value	0.006	0.001	<0.001	

^a^ ^,b,c,d,A,B^Mean values followed by the same lowercase letters along the columns and uppercase letters along the rows did not statistically differ based on Tukey’s test (*p* > 0.05).

Although the 2D group showed the second highest concentration of EVs, there were no statistical differences with the other groups at 24 h and 120 h. Interestingly, a moderate but significant decrease of approximately 25% in the concentration of EVs was observed at 72 h in the 2D group, whereas the 3D group demonstrated a severely decreased concentration of EVs at 72 h (approximately 50%), which was statistically lower than its value at the 24 h time point (*p* < 0.001). On the other hand, the 2D-OA group showed similar results at all time points (*p* = 0.210). Comparison of the mean EV sizes at 24 h revealed smaller EVs in groups 3D and 3D-OA, which were statistically different from that of the 2D-OA group (*p* = 0.006). At 72 h, group 2D showed larger EVs, which was statistically different from all the other groups (*p* = 0.001). At 120 h, the 3D-OA group showed smaller EV sizes, and all groups presented with significant differences (*p* < 0.001), with larger sizes of EVs in both the control groups.

Considering each group individually, the 2D group showed an increase of approximately 44% in the mean EV size between 24 h and 72 h, followed by a slight decrease at 120 h. At the 24 h time point, statistical differences were observed with the other time points for this group (*p* = 0.006). The 3D group showed a progressive enhancement of approximately 27% in the mean EV size through the experimental time points, presenting statistical differences from 24 h to 120 h (*p* = 0.010). The 2D-OA and 3D-OA groups showed decreasing mean EV sizes through the time points ( 12% and 11%, respectively), without any statistical significance (*p* = 0.182 and *p* = 0.316, respectively).

### Gene expressions for characterization of the EVs

Relative mRNA expressions of the tetraspanins *CD9*, *CD63*, and *CD81 versus* normalized control *GAPDH* were heterogeneously observed in all groups at all time points, confirming the feasibility of EV characterization.

#### CD9

**FIGURE 5:**
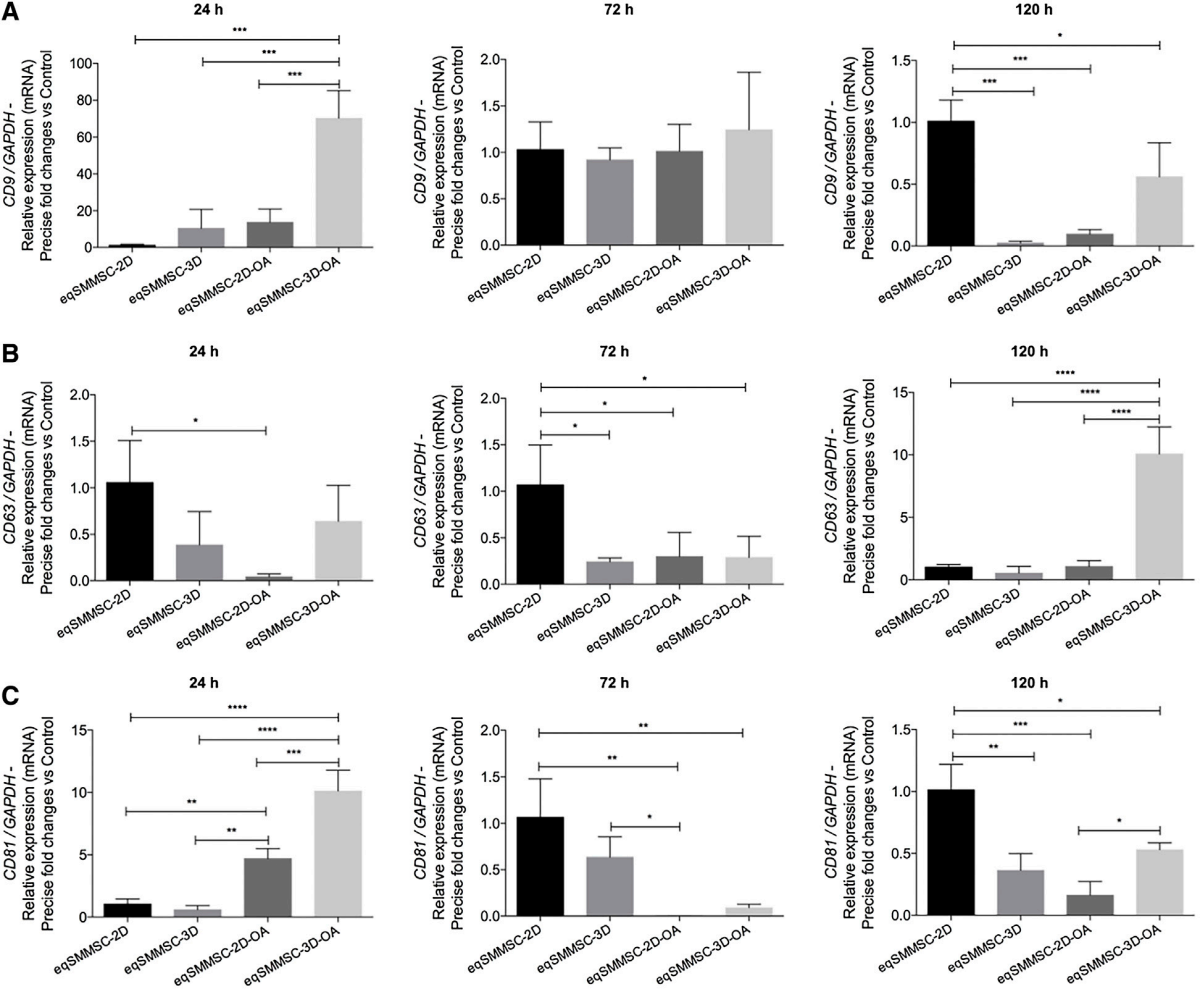
EV characterization by the relative gene expressions of tetraspanins CD9, CD63, and CD81, with normalization of GAPDH. *p* values: *p* ≤ 0.05*, *p* ≤ 0.01**, *p* ≤ 0.001***, and *p* ≤ 0.0001****.

The expression of *CD9* was higher at 24 h in the 3D-OA group compared to all other groups. This expression decreased significantly at 72 h, at which point there were no statistical differences among the groups. At 120 h, the 2D group maintained its expression, whereas the 3D and 2D-OA groups showed decreased expressions compared to the other groups. At this time point, *CD9* expression in the 3D-OA group decreased significantly, differing from those of the 3D (*p* = 0.0165) and 2D-OA (*p* = 0.0343) groups (Figure 5A). Considering each group in isolation, there was a pattern of decrease in *CD9* expression through time. Groups 2D and 3D did not present differences through time, whereas the treatment groups 2D-OA and 3D-OA showed statistical differences between 24 h and 120 h (*p* = 0.027 and *p* < 0.001, respectively). These individual group results are shown in Supplementary Data S3.

#### CD63

At 24 h, the 2D group demonstrated a significantly higher expression of *CD63* compared to the 2D-OA group (*p* = 0.0292). The higher expression in the 2D group was significantly superior to all the groups at 72 h. Even though the 2D group maintained a high expression of *CD63* at 120 h, the 3D-OA group presented a significant increase over the other groups (*p* < 0.0001) (Figure 5B). The mean *CD63* expressions within the groups showed minimal variations through time. However, the treated groups showed progressively increased expressions, with differences between 24 h and 120 h in the 2D-OA group (*p* = 0.028), whereas the expression of the 3D-OA group at the 120 h time point was significantly higher than those at 24 h and 72 h (*p* = 0.001). These individual group results are shown in Supplementary Data S4.

#### CD81

The 2D-OA and 3D-OA treatment groups showed higher expressions at 24 h compared to the control groups, with the highest expression occurring in the 3D-OA group compared to the 2D-OA group. Significant decreases were observed at 72 h in the 2D-OA and 3D-OA groups, whereas both control groups had maintained expressions. Furthermore, the 2D group showed a statistically higher expression than the 2D-OA (*p* = 0.0025) and 3D-OA (*p* = 0.0042) groups, and there was a difference between the 3D and 2D-OA (*p* = 0.0451) groups. Group 2D was significantly different from all groups at 120 h, and there was higher expression in the 3D-OA group compared to the 2D-OA (*p* = 0.0455) group (Figure 5C). Individually, the *CD81* expressions varied only in the 2D-OA and 3D-OA groups between 24 and 72 h (both *p* < 0.001). These individual group results are shown in Supplementary Data S5.

### Expression of microRNA-140

**FIGURE 6:**
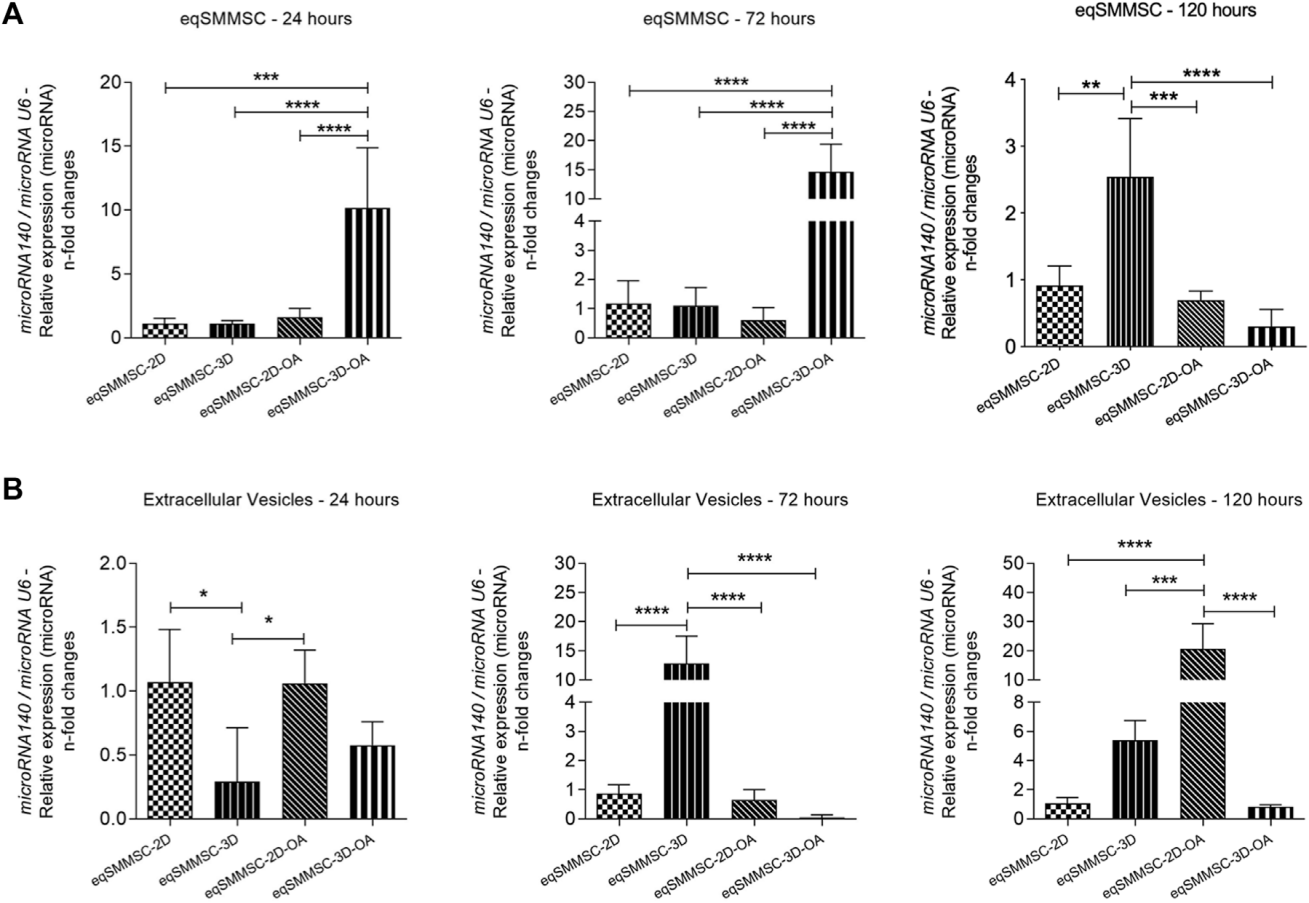
Analysis of microRNA-140 relative expressions in eqSMMSCs and EVs, with normalization of GAPDH. **(A)** shows the expression of eqSMMSCs and **(B)** shows EV expressions at different time points. *p* values: *p* ≤ 0.05*, *p* ≤ 0.01**, *p* ≤ 0.001***, and *p* ≤ 0.0001****.

The expressions of microRNA-140 between eqSMMSCs and EVs were observed at different levels in all groups at all time points, predominantly in groups 2D-OA, 3D, and 3D-OA (Figure 6).

#### eqSMMSCs

The expression of microRNA-140 varied with time, with a 7.5-fold increased expression in the 3D-OA group than the control groups 2D (*p* = 0.0001) and 3D (*p* < 0.0001) as well as a 7-fold increase compared to group 2D-OA (*p* < 0.0001) at 24 h. At 72 h, the 3D-OA group maintained a higher expression compared to the other groups, which was 14-fold higher than the 2D (*p* < 0.0001) and 3D (*p* < 0.0001) groups as well as 15-fold higher than the 2D-OA group (*p* < 0.0001). At 120 h, however, decreases in expressions were noted in all the groups, with similar expressions for all groups. At this time point, the 3D group presented a slight increase over the other groups, with 1.2-fold higher values than the 2D (*p* = 0.0014) and 2D-OA (*p* = 0.0005) groups as well as 1.7-fold higher value than the 3D-OA (*p* < 0.0001) group (Figure 6A). Independently, differences were observed in the 2D-OA group between 24 h and 72 h (*p* = 0.029), and the 3D-OA group showed differences between 72 h and 120 h (*p* = 0.006), with no differences being observed in the control groups. These individual group results are shown in Supplementary Data S6.

#### EVs

Relative expressions of the microRNA-140 in the EVs presented differences from the cell expressions, with the values being lower in the EVs. Differences were observed between the groups at 24 h, with the lowest expression occurring in the 3D group, with was 0.7-fold lower than those of the 2D (*p* = 0.0174) and 2D-OA (*p* = 0.0187) values. At 72 h, while the 2D, 2D-OA, and 3D-OA groups demonstrated lower expressions of microRNA-140, group 3D showed a significant increase of approximately five times in its expression. There was a significant difference in the 3D group when compared to all other groups (*p* < 0.0001). At 120 h, group 3D showed a discrete decrease in its expression, whereas the 2D-OA group showed a significant increase and statistical differences with all other groups (*p* < 0.0001); this was 15 times higher than that of the 3D (*p* = 0.0006) group and 20 times higher than those of the 2D and 3D-OA (*p* < 0.0001) groups (Figure 6B). Considering each group in isolation, only the treatment groups presented statistically significant increases between 72 h and 120 h (2D-OA with *p* = 0.006 and 3D-OA with *p* < 0.001). These individual group results are shown in Supplementary Data S7. No further differences were observed.

#### Correlation of microRNA-140 in eqSMMSCs and EVs

**FIGURE 7:**
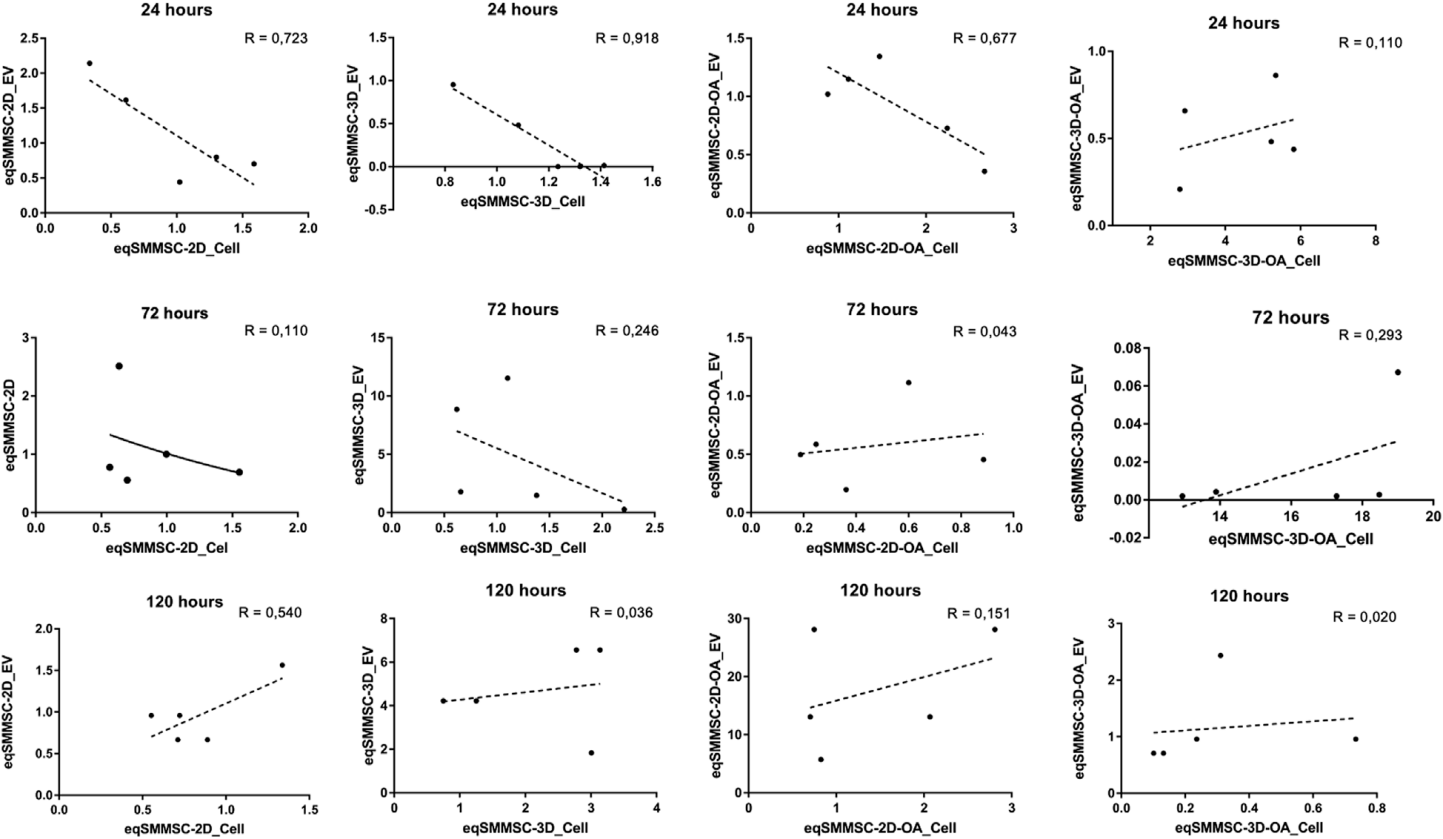
Analysis of the data correlation coefficient for microRNA-140 expressions between eqSMMSCs and EVs for the different groups at different times. R-squared values are related to data dispersion and quality of the sample N. P is the Pearson’s correlation coefficient.

Negative correlations were observed at 24 h in the 2D (EVs *versus* cells; *p* = −0.851), 3D (EVs *versus* cells; *p* = −0.958), and 2D-OA (EVs *versus* cells; *p* = −0.823) groups. The analysis of groups at the remaining time points revealed positive correlations for the 3D-OA group at 72 h and 120 h (*p* = 0.616 and *p* = 0.791, respectively) time points, where it was possible to observe concomitant decreases in the expressions of microRNA-140 in both cells and EVs (Figure 7).

### Gene expression of *Adamts5* within the EVs

**FIGURE 8:**
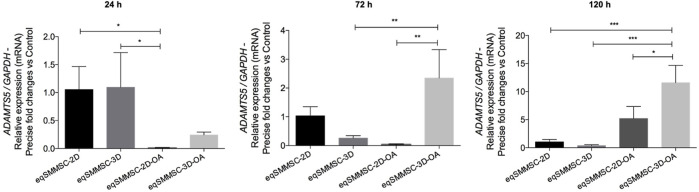
*Adamts5* relative gene expression analysis in EVs with normalization of GAPDH. *p* values: *p* ≤ 0.05*, *p* ≤ 0.01**, and *p* ≤ 0.001***.

Results of the *Adamts5* gene expressions are shown in Supplementary Data S8 and Figure 8. A significant difference (*p* = 0.0144) was observed at 24 h between the control and treatment groups. At 72 h, the expression was maintained in the 2D group but decreased in the 3D and significantly increased in the 3D-OA groups, with 2-times higher expression than the 3D (*p* = 0.0051) and 2.3-times higher expression than the 2D-OA (*p* = 0.0027) groups. At 120 h, the 3D-OA group showed a notable increase in *Adamts5* gene expression compared to the other groups (*p* < 0.0003), with 2-times increase compared to the 2D-OA (*p* = 0.0136), 10.5 times increase than the 2D (*p* = 0.0006), and 11 times increase than the 3D (*p* = 0.0004) groups. Individually, only the treatment groups showed differences over time. Group 2D-OA and 3D-OA showed increased expressions at 120 h than at 24 h and 72 h (*p* = 0.010 and *p* = 0.006, respectively).

## Discussion

Several factors, including the environment and experimental model, can influence the release of EVs and their contents during signaling by the SMMSCs. In the context of our study, we observed no significant signs of cell damage or death in the 3D-OA group, suggesting that these cells were resilient to the inflammatory environment (Sun et al., 2011). This resilience could be attributed to two main factors, namely, the three-dimensional organization of the MSCs in the sodium alginate scaffold (which potentially enhances their paracrine functions by mimicking natural cell arrangements to facilitate cellular interactions) and the physical barrier provided by the scaffold itself (which may protect cells from inflammatory proteins and other detrimental factors present in the OA-like medium) (Lee and Mooney, 2012).

The gradual and indirect exposure of the cells to the inflammatory environment may have prompted their ability to become primed and adapt to the conditions of OA (Barrachina et al., 2017; Cassano et al., 2018b; Lange-Consiglio et al., 2020). This adaptation could potentially explain the superior outcomes observed in the 3D-OA group. Furthermore, alginate has the capability to bind low-molecular-weight substances, such as cytokines (Lee and Mooney, 2012), which could reduce their concentration in the medium and facilitate cell priming.

The fluctuation in the EV concentrations may be a result of various interactions among the cells, scaffolds, and culture medium in the three-dimensional cultures or from high cell confluence in the 2D monolayer group. Inflammatory conditions, such as those simulated in this study, significantly impact cell behaviors and secretion profiles. Moreover, the EVs serve as crucial carriers of intercellular communications (Zech et al., 2012; Mulcahy et al., 2014; Van Niel et al., 2018) and are continuously produced and absorbed within a normal cell population. Their production and release are intricately linked to cellular responses and environmental cues, underscoring their importance in cell signaling and regulatory mechanisms.

Higher concentrations of EVs were initially observed in the three-dimensional groups, which is consistent with findings from an earlier study comparing monolayer and spheroid cultures of murine colorectal cancer cells (Hwang et al., 2019). Inflammation appears to play a pivotal role in EV production, as demonstrated in a study by Viñuela-Berni et al. (2015), which reported increased levels of EVs in urine and blood samples from patients with lupus or rheumatoid arthritis; this study also showed that EVs stimulated the release of TNF-α and IL-1 *in vitro*. MSCs also show increased EV production when exposed to media containing IFN-γ (Ragni et al., 2020). Similarly, cytokines used in the OA-like medium (IL-1β and TNF-α) may have influenced the concentration of EVs in the 3D-OA group.

High cell confluence in the monolayer culture may have also contributed to the increase in EVs over time, as the cells continue to proliferate within a confined and limited culture area. In contrast, the absence of protective and attenuating mechanisms in the 2D-OA group likely resulted in cell death and a subsequent decrease in EVs over time compared to the control 2D group. This occurred despite the fact that the combination of IL-1β and TNF-α at the concentrations used in this study is considered optimal for an *in vitro* experimental model of OA (Johnson et al., 2016).

It is plausible that the sizes of the EVs were overestimated in this study owing to the precipitation method used, which tends to form EV aggregates. This can result in overestimation when analyzing EV size by NTA (Klymiuk et al., 2019). Our findings on the EV sizes are aligned with those reported by Patel et al., who also investigated various EV isolation methods (Patel et al., 2019). Therefore, the mean size obtained through NTA may not correspond with the results from TEM, where the EVs typically appeared to be around 100 nm in size. This discrepancy suggests the possibility of EV aggregation during the precipitation process.

The presence of transmembrane proteins such as CD9, CD63, and CD81 is crucial for accurately characterizing the EVs and confirming the contents of the samples (Welsh et al., 2024). Tetraspanins like CD9 and CD81 play roles in selecting the cargo molecules transported by EVs, and they are involved in regulating the endosomal sorting complex required for transport (ESCRT) machinery (Chairoungdua et al., 2010). On the other hand, CD63 is directly associated with the synthesis of EVs, which may occur through ESCRT-independent pathways (Van Niel et al., 2018).

According to the International Society of Extracellular Vesicles, the presence of tetraspanins such as CD9, CD63, and CD81 should ideally be confirmed using methods like ELISA, colorimetric analysis, or flow cytometry. These techniques utilize beads or cytometers capable of detecting nanoparticles (Welsh et al., 2024). However, gene expression analysis can also provide valuable insights into the presence and expression levels of tetraspanins. It has been observed that gene expression analysis can reveal heterogeneous expression patterns of tetraspanins among different experimental groups over time. The influence of the OA-like medium should indeed be considered, as evidenced by the higher expressions of CD9 and CD81 observed in the 3D-OA and 2D-OA groups. This suggests that the inflammatory medium may have induced a significant increase in the selection of cargo molecules within the EVs via CD9 and CD81. These tetraspanins are known to play crucial roles in regulating the cargo and biogenesis of EVs, potentially modulating the cellular environment through the eqSMMSCs.

CD63 is known to be involved in the biogenesis of EVs, particularly in the process where intraluminal vesicles are formed within multivesicular bodies (MVBs) before being released as exosomes (Van Niel et al., 2018). The higher expression of CD63 observed in the 3D-OA group at 120 h may indeed be related to the smaller size of the EVs observed in the NTA. This finding is aligned with the results of the study by Edgar et al. (2014), which noted a correlation between CD63 expression and formation of small intraluminal vesicles, often referred to as pre-exosomes. The higher expression of CD63 in the 3D-OA group suggests an active process of EV formation, potentially leading to the production of smaller EVs, as detected by the NTA. These insights underscore the dynamic nature of EV biogenesis and regulatory roles played by tetraspanins like CD63 in responding to inflammatory conditions, such as those simulated in the OA-like medium.

The expressions of microRNA-140 in both the EVs and eqSMMSCs are indicative of paracrine signaling, reflecting its crucial role in maintaining articular homeostasis (Miyaki et al., 2009; Tardif et al., 2009; Miyaki et al., 2010; Karlsen et al., 2016; Tao et al., 2017; Yin et al., 2017). The results from both control groups in our study exhibited normal microRNA-140 expressions, which are aligned with previous findings in healthy joints (Yin et al., 2017). This consistency suggests that under normal physiological conditions, microRNA-140 is appropriately regulated and contributes to the maintenance of joint health and function.

Yin et al. (2017) demonstrated an inverse relationship between the expression of microRNA-140 in human synovial fluid and severity of OA, reporting a relative expression (fold-change) of 0.36 in mild cases, 0.173 in moderate cases, and 0.036 in severe cases, compared to 1.08 in the control group. In our study, we observed 7-fold and 14-fold increases in the relative expressions of microRNA-140 in eqSMMSCs from the 3D-OA group at 24 h and 72 h, respectively, after exposure to an inflammatory environment. These findings suggest that modulation of the microRNA-140 expressions in the eqSMMSCs under inflammatory conditions may parallel observations in human OA.

The increase in the expression of microRNA-140 observed at 120 h in the 3D group may indeed be influenced by the extended culture period, as suggested by Lange-Consiglio et al. (2018b). Their study demonstrated alterations in the microRNA profiles of amniotic stem cell cultures beyond 96 h, indicating potential phenotypic changes and senescence. Additionally, chondrogenesis induced by the alginate scaffold (Santos et al., 2018) might have contributed to the observed changes in microRNA expression in our study. It is known that microRNA-140 can be activated by the transcription factor SRY-box 9 (*SOX9*), and there is a positive correlation between microRNA-140 expression and *SOX9* during embryonic chondrogenesis and chondrogenic differentiation of MSCs (Nakamura et al., 2011; Nakamura et al., 2012; Buechli et al., 2013; Tao et al., 2017). Therefore, the combination of extended culture duration and chondrogenic induction by the scaffold could have synergistically influenced the expressions of microRNA-140 in our experimental setup.

The high expression of microRNA-140 observed in the MSCs of the 3D-OA group contrasted with its discrete expression within the EVs, suggesting that MSCs synthesized microRNA-140 robustly while only a small portion of it was transported via the EVs. This discrepancy may reflect the selective packaging mechanisms or regulatory processes within the cells, where not all synthesized microRNAs are encapsulated into EVs for extracellular communication. Conversely, the delayed response in microRNA-140 expression observed in the 2D-OA group could be attributed to initial cell damage caused by direct contact with the inflammatory medium. This damage may have temporarily disrupted cellular processes, including the synthesis and release of microRNAs via the EVs. Overall, our results suggest that the microRNA-140 production is mobilized in response to inflammatory conditions in an effort to restore homeostasis. This is supported by the observation that both groups exposed to the inflammatory medium (2D-OA and 3D-OA) expressed microRNA-140 in the MSCs as well as in EVs.

The finding that both MSCs and EVs expressed microRNA-140 under inflammatory conditions is contrary to the initial observations by Miyaki et al. (2009), who reported downregulation of microRNA-140 and upregulation of ADAMTS5 in chondrocytes treated with IL-1β. However, an important subsequent step in their study demonstrated that transfection of microRNA-140 effectively suppressed the expression of ADAMTS5 in chondrocytes exposed to IL-1β, thereby reinforcing the regulatory role of microRNA-140 in chondral metabolism. Importantly, while inflammatory stimuli like IL-1β can initially downregulate endogenous microRNA-140, the cellular responses may involve compensatory mechanisms to restore its expression. This could explain why we observed increased microRNA-140 expressions in the MSCs and EVs under inflammatory conditions in our study, potentially reflecting cellular responses to counterbalance inflammation and restore homeostasis.

The mechanical protection provided by the sodium alginate capsules and observed expressions of microRNA-140 in the 3D-OA group are aligned with findings by He et al. (2020), who reported a positive influence of the exosomes secreted by BMMSCs on *in vitro* cartilage repair models. In their study, chondrocytes that internalized exosomes via endocytosis showed reduced detrimental effects of IL-1β on cell proliferation and migration. Similarly, our results demonstrate lower deleterious impacts of the inflammatory medium on cells cultured in three-dimensional environments.

While the expression of *Adamts5* was not specifically assessed in the MSCs in our study, its presence in the EVs from both control groups suggests that the cells indeed expressed this gene. ADAMTS5 is a physiological enzyme involved in the maintenance of tissue balance between catabolism and anabolism, and it is normally released under homeostasis conditions. For instance, chondrocytes have been observed to express significantly more ADAMTS5 than BMMSCs during early chondrogenic differentiation (Boeuf et al., 2012). Additionally, the expression of ADAMTS5 can vary across different species, cell types, and experimental conditions (Fosang et al., 2008). However, dysregulated expression of *Adamts5* can lead to a catabolic state, disrupting tissue homeostasis. The higher expressions observed in both control groups after exposure to inflammatory stimuli reinforce the association between inflammation and synthesis of ADAMTS5, particularly under prolonged exposure to inflammatory cytokines.

The characterizations and comparisons of EV synthesis and microRNA-140 expression in both 2D and 3D cell cultures represent significant achievements. These findings contribute to our understanding of the roles of MSCs in the treatment of inflammatory injuries, such as OA. Overall, these findings pave the path for further research and development of MSC-based therapies targeted at inflammatory injuries, including OA. They underscore the importance of the culture methods and environment in harnessing the therapeutic potentials of MSC-derived EVs and microRNAs.

Future studies focusing on the paracrine signaling pathways of SMMSCs in OA, particularly through their secretion of EVs, expression of microRNAs, and activation of enzymes, hold promise in the development of novel targeted therapies aimed at halting OA progression or restoring functional cartilage surfaces.

## Conclusion

Overall, our findings validate the efficacy of the experimental model for isolating EVs and studying microRNA-140 expressions. Importantly, inflammation significantly influences various aspects of cell behaviors, including morphology, proliferation, and gene expression profiles, in eqSMMSCs. Depending on the stimulus and cell culture conditions, eqSMMSCs synthesize EVs containing different concentrations of microRNA-140 in response. Despite the limitation of this study that considers only one cell donor and therefore lacks biological replication, we highlight that cells cultured in a 3D scaffold of sodium alginate exhibit prompt responses to inflammatory stimuli by upregulating microRNA-140, indicating their roles in seeking to restore homeostasis within the environment. In contrast, cells in the monolayer culture demonstrate delayed responses, underscoring the potential protective effects conferred by the 3D scaffold.

The discrepancies observed between the results from different culture models emphasize the necessity of exploring experimental methods that are closer to the *in vivo* physiological conditions. This approach is crucial for advancing both animal and human studies of OA toward clinical applications.

## Figures and Tables

**Table:** 

**2D**	Bidimensional
**3D**	Tridimensional
**˚C**	Celsius degrees
**CaCl**_**2**_	Calcium chloride
**h**	Hour
**min**	Minute
**mL**	Milliliter
**ng**	Nanogram
**µm**	Micrometer
**µL**	Microliter
**×*g***	Relative centrifugal force
**ADAMTS-5**	A disintegrin and metalloprotease with thrombospondin type 5
**CD**	Cluster of differentiation
**cDNA**	Complementary deoxyribonucleic acid
**CEUA**	Ethics committee on animal use
**DMEM/F-12**	Dulbecco’s modified Eagle medium: nutrient mixture F12
**DNA**	Deoxyribonucleic acid
**eqSMMSCs**	Equine synovial-membrane-derived mesenchymal stem cells
**EVs**	Extracellular vesicles
**FBS**	Fetal bovine serum
**FBSevfree**	Fetal bovine serum free of extracellular vesicles
**GAPDH**	Glyceraldehyde 3-phosphate dehydrogenase
**IL-1β**	Interleukin-1 beta
**mRNA**	Messenger ribonucleic acid
**MSC**	Mesenchymal stem cell
**NTA**	Nanoparticle tracking analysis
**OA**	Osteoarthritis
**PBS**	Phosphate buffer solution
**RT-qPCR**	Reverse transcription quantitative polymerase chain reaction
**SD**	Standard deviation
**TEM**	Transmission electron microscopy
**TNF*α***	Tumor necrosis factor alpha

## Data Availability

The datasets presented in this study can be found in online repositories. The names of the repository/repositories and accession number(s) can be found in the article/Supplementary Material.

## Supplementary material

SUPPLEMENTARY DATA S1 Transmission electron microscopy of FBS*evfree*.

SUPPLEMENTARY DATA S2 Sequences of the target and internal control primers for the gene expressions, and cycle conditions (45) of the RT-qPCR.

SUPPLEMENTARY DATA S3 CD9 expressions on the EVs. The data are presented in terms of mean±SD.

SUPPLEMENTARY DATA S4 CD63 expressions on the EVs. The data are presented in terms of mean±SD.

SUPPLEMENTARY DATA S5 CD81 expressions on the EVs. The data are presented in terms of mean±SD.

SUPPLEMENTARY DATA S6 eqSMMSC microRNA-140 expression. The data are presented in terms of the median as well as 25th and 75th percentiles.

SUPPLEMENTARY DATA S7 Expression of microRNA-140 in the EVs. The data are presented in terms of the median as well as 25th and 75th percentiles.

SUPPLEMENTARY DATA S8 Relative gene expression of *Adamts5* in the EVs. The data are presented in terms of the mean±SD.
