# Supplementary material for: Influence of inflammation on the expression of microRNA-140 in extracellular vesicles from 2D and 3D culture models of synovial-membrane-derived stem cells
Source: Front Bioeng Biotechnol. 2024 Aug 7;12:1416694. doi: 10.3389/fbioe.2024.1416694 (PMC11335645; doi:10.3389/fbioe.2024.1416694)
Supplement: Supplementary file 1 [file DataSheet7.PDF]

Supplementary data 7. EVs microRNA-140 expression. The data are presented by median and percentile 25 and 75%.

**Groups**	**Time Points**			**P**
	**24h**	**72h**	**120h**	
**2D**	0,98 [0,70 – 1,62] aA	0,89 [0,69 – 1,32] bA	0,96 [0,67 -1,56] bA	**0,311**
**3D**	0,25 [0,00 – 0,95] aA	5,32 [1,48 – 11,53] aA	4,22 [1,84 – 6,56] abA	**0,184**
**2D-OA**	0,87 [0,36 – 1,15] aB	0,54 [0,46 – 1,03] bB	13,09 [5,74 – 28,13] aA	**0,006**
**3D-OA**	0,46 [0,44 – 0,66] aB	0,00 [0,00 – 0,007] bB	0,96 [0,71 – 2,44] bA	**<0,001**
**P**	**0,236**	**0,001**	**<0,001**	

* Median followed by the same lowercase letter on columns and uppercase letter on lines did not statistically differ by Tukey's test (P>0,05).
