# Supplementary material for: Influence of inflammation on the expression of microRNA-140 in extracellular vesicles from 2D and 3D culture models of synovial-membrane-derived stem cells
Source: Front Bioeng Biotechnol. 2024 Aug 7;12:1416694. doi: 10.3389/fbioe.2024.1416694 (PMC11335645; doi:10.3389/fbioe.2024.1416694)
Supplement: Supplementary file 2 [file DataSheet2.PDF]

Supplementary data 2. Sequence of target and internal control primers for gene expression, and cycle conditions (45) of RT-qPCR reaction.

Gene	Primer	Sequence 5'-3'	Condition
CD9	Forward	CAAGCTTCACAACGAAGCCC	95°C - 20s; 95°C - 01s; 60°C - 20s.
	Reverse	GCGCCGATGATGTGGAATTT	
CD63	Forward	GTTCTTCTGCTGGCCTTTTGC	
	Reverse	CTGAGTGACATTGACGCAGC	
CD81	Forward	CCTGCCTGGTGATCCTGTTT	
	Reverse	GGCGATCTGGTCCTTGTTGA	
Adamts5	Forward	TAGAGGCATGGCTTGTCGTG	
	Reverse	AAGGTGTGGTAGAAGGCGTG	
GAPDH	Forward	TCAGCCATCGCCAAGATTGT	
	Reverse	CCCGACGTCTTCCTCAAACA	
