# Supplementary material for: Influence of inflammation on the expression of microRNA-140 in extracellular vesicles from 2D and 3D culture models of synovial-membrane-derived stem cells
Source: Front Bioeng Biotechnol. 2024 Aug 7;12:1416694. doi: 10.3389/fbioe.2024.1416694 (PMC11335645; doi:10.3389/fbioe.2024.1416694)
Supplement: Supplementary file 3 [file DataSheet4.PDF]

Supplementary data 4. CD63 expression on EVs. Data are presented by mean±SD.

**Groups**	**Time Points**			**P**
	**24h**	**72h**	**120h**	
**2D**	1,06 ± 0,45 aA	1,07 ± 0,43 aA	1,02 ± 0,22 bA	**0,986**
**3D**	0,38 ± 0,36 abA	0,24 ± 0,04 bA	0,54 ± 0,54 bA	**0,717**
**2D-OA**	0,04 ± 0,03 bB	0,30 ± 0,26 bAB	1,07 ± 0,48 bA	**0,028**
**3D-OA**	0,64 ± 0,39 abB	0,29 ± 0,23 bB	10,07 ± 2,17 aA	**0,001**
**P**	**0,038**	**0,017**	**<0,001**	

* Means followed by the same lowercase letter on columns and uppercase letter on lines did not statistically differ by Tukey’s test (P>0,05).
