# Supplementary material for: Influence of inflammation on the expression of microRNA-140 in extracellular vesicles from 2D and 3D culture models of synovial-membrane-derived stem cells
Source: Front Bioeng Biotechnol. 2024 Aug 7;12:1416694. doi: 10.3389/fbioe.2024.1416694 (PMC11335645; doi:10.3389/fbioe.2024.1416694)
Supplement: Supplementary file 4 [file DataSheet6.PDF]

Supplementary data 6. eqSMMSCs microRNA-140 expression. The data are presented by median and percentile 25 and 75%.

**Groups**	**Time Points**			**P**
	**24h**	**72h**	**120h**	
**2D**	1,16 [0,61 – 1,59] bA	0,85 [0,63 -1,55] bA	0,80 [0,71 – 1,34] aA	**0,956**
**3D**	1,16 [0,86 – 1,32] bA	0,89 [0,66 – 1,38] bA	2,01 [0,75 – 3,01] aA	**0,740**
**2D-OA**	1,38 [1,11 – 2,24] bA	0,48 [0,25 – 0,88] bB	0,79 [0,70 – 2,07] aAB	**0,029**
**3D-OA**	8,49 [5,22 – 12,79] aA	15,58 [12,95 – 18,48] aA	0,27 [0,13 – 0,73] aB	**0,006**
**P**	**0,003**	**0,002**	**0,076**	

* Median followed by the same lowercase letter on columns and uppercase letter on lines did not statistically differ by Tukey's test (P>0,05).
