# Supplementary material for: Influence of inflammation on the expression of microRNA-140 in extracellular vesicles from 2D and 3D culture models of synovial-membrane-derived stem cells
Source: Front Bioeng Biotechnol. 2024 Aug 7;12:1416694. doi: 10.3389/fbioe.2024.1416694 (PMC11335645; doi:10.3389/fbioe.2024.1416694)
Supplement: Supplementary file 5 [file DataSheet3.PDF]

Supplementary data 3. CD9 expression on EVs. Data are presented by mean±SD.

Groups	Time Points			P
	**24h**	**72h**	**120h**	
**2D**	1,11 ± 0,61 bA	1,03 ± 0,30 aA	1,01 ± 0,17 aA	**0,952**
**3D**	10,47 ± 10,28 bA	0,92 ± 0,13 aA	0,02 ± 0,02 bA	**0,172**
**2D-OA**	13,71 ± 7,27 bA	1,01 ± 0,29 aB	0,10 ± 0,04 bB	**0,027**
**3D-OA**	70,19 ± 15,02 aA	1,24 ± 0,62 aB	0,56 ± 0,28 aB	**<0,001**
**P**	**<0,001**	**0,762**	**<0,001**	

* Means followed by the same lowercase letter on columns and uppercase letter on lines did not statistically differ by Tukey's test (P>0,05).
