# Supplementary figures and images for: Influence of inflammation on the expression of microRNA-140 in extracellular vesicles from 2D and 3D culture models of synovial-membrane-derived stem cells
Source: Front Bioeng Biotechnol. 2024 Aug 7;12:1416694. doi: 10.3389/fbioe.2024.1416694 (PMC11335645; doi:10.3389/fbioe.2024.1416694)

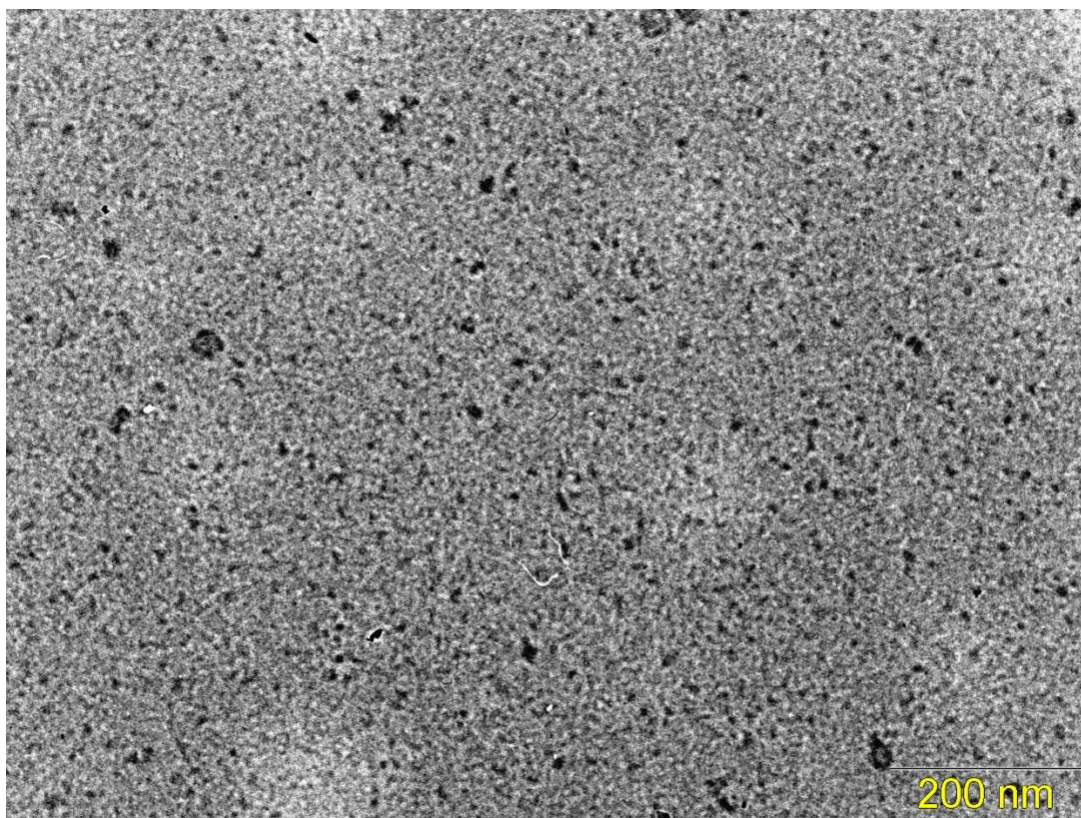

**Supplementary data 1.** Transmission Electron Microscopy from the FBSevfree.

Supplement: Supplementary file 6 [file DataSheet1.PDF]
