# Supplementary material for: Influence of inflammation on the expression of microRNA-140 in extracellular vesicles from 2D and 3D culture models of synovial-membrane-derived stem cells
Source: Front Bioeng Biotechnol. 2024 Aug 7;12:1416694. doi: 10.3389/fbioe.2024.1416694 (PMC11335645; doi:10.3389/fbioe.2024.1416694)
Supplement: Supplementary file 7 [file DataSheet5.PDF]

Supplementary data 5. CD81 expression on EVs. Data are presented by mean±SD.

**Groups**	**Time Points**			**P**
	**24h**	**72h**	**120h**	
**2D**	1,06 ± 0,41 cA	1,07 ± 0,41 aA	1,01 ± 0,21 aA	**0,985**
**3D**	0,60 ± 0,34 cA	0,63 ± 0,22 abA	0,36 ± 0,14 bcA	**0,462**
**2D-OA**	4,70 ± 0,80 bA	0,00 ± 0,00 bcB	0,16 ± 0,11 cB	**<0,001**
**3D-OA**	10,12 ± 1,66 aA	0,09 ± 0,04 bB	0,53 ± 0,06 bB	**<0,001**
**P**	**<0,001**	**0,002**	**<0,001**	

* Means followed by the same lowercase letter on columns and uppercase letter on lines did not statistically differ by Tukey's test (P>0,05).
