# Supplementary material for: Influence of inflammation on the expression of microRNA-140 in extracellular vesicles from 2D and 3D culture models of synovial-membrane-derived stem cells
Source: Front Bioeng Biotechnol. 2024 Aug 7;12:1416694. doi: 10.3389/fbioe.2024.1416694 (PMC11335645; doi:10.3389/fbioe.2024.1416694)
Supplement: Supplementary file 8 [file DataSheet8.PDF]

Supplementary data 8. EVs relative gene expression of *Adamts5*. Data presented by mean±SD.

**Groups**	**Time Points**			**P**
	**24h**	**72h**	**120h**	
**2D**	1,05 ± 0,41 aA	1,04 ± 0,31 bA	1,05 ± 0,42 bA	**0,998**
**3D**	1,10 ± 0,62 aA	0,26 ± 0,08 bA	0,37 ± 0,18 bA	**0,140**
**2D-OA**	0,01 ± 0,00 bB	0,04 ± 0,02 bB	5,22 ± 2,12 bA	**0,010**
**3D-OA**	0,24 ± 0,05 bB	2,35 ± 0,99 aB	11,60 ± 3,08 aA	**0,006**
**P**	**0,014**	**0,002**	**<0,001**	

* Mean followed by the same lowercase letter on columns and uppercase letter on lines did not statistically differ by Tukey’s test (P>0,05).
